# Impact of age on functional recovery following hospital-based rehabilitation in older adults

**DOI:** 10.1007/s11739-025-04219-4

**Published:** 2025-12-14

**Authors:** Alessandra Petrelli, Tiziano Nestola, Cesare Mainetti, Sarah Catarame, Francesca Giani, Giovanna Esposti, Gessica Nicodemi, Calogero Malfitano, Paolo Fiorina, Francesco Dentali, Stefania Iacono, Nadia Antoniotti, Sonia Baruffi, Antonella Ferrari, Marco Froldi

**Affiliations:** 1 Department of Clinical Sciences and Community Health, Department of Excellence 2023-27, University of Milan, Via Festa del Perdono, 7, 20122 Milano, Italy; 2 Pio Albergo Trivulzio, Milan, Italy; 3 International Center for T1D, Pediatric Clinical Research Center Romeo Ed Enrica Invernizzi, DIBIC, Università Degli Studi Di Milano, Milan, Italy; 4 School of Geriatrics, University of Milan, Milan, Italy; 5 Department of Biomedical Sciences for Health, University of Milan, Milan, Italy; 6 Division of Endocrinology, ASST Fatebenefratelli-Sacco, Milan, Italy; 7 Nephrology Division, Boston Children’s Hospital, Boston, MA USA; 8 Università Degli Studi Dell’Insubria, Varese, Italy

**Keywords:** Frailty, Geriatric assessment, Inpatients, Recovery of function, Rehabilitation, Aged

## Abstract

With a rapidly aging population, accurately predicting rehabilitation outcomes in older adults is increasingly important. As the impact of age on recovery trajectories in geriatric rehabilitation remains unclear, we conducted a retrospective observational study to evaluate the effect of older age on improvements in rehabilitation functional outcomes. We included 158 patients (median age: 84 years; IQR: 77.0–88.0) admitted in 2024 to a geriatric rehabilitation unit. Functional status was assessed at admission and discharge using the Modified Barthel Index (autonomy), Hendrich II Fall Risk Model (fall risk), and Tinetti Scale (balance and gait). Patients were stratified into age tertiles and compared using the Kruskal–Wallis and Mann–Whitney U tests. Multivariable linear regression models evaluated the independent associations of age, sex, and frailty index with functional outcomes. Comparisons of changes in functional outcomes between the youngest (T1) and oldest (T3) age tertiles were performed using the Mann–Whitney U test. Older age was associated with worse baseline functional status, reflected by lower Barthel and Tinetti scores and higher Hendrich II scores. However, in multivariable models, frailty index—but not chronological age—was the strongest independent predictor of all functional outcomes, including Barthel (*β* =  −89.9, *p* < 0.001), Tinetti (*β* =  −39.9, *p* < 0.001), and Hendrich II (*β* = 6.8, *p* < 0.001). Both younger and older patients achieved significant functional gains, particularly in mobility, whereas improvements in autonomy and fall risk were more limited among the oldest individuals. In conclusion, chronological age was associated with poorer baseline performance but did not impair motor recovery. Frailty, rather than age itself, emerged as the key determinant of rehabilitation outcomes. These findings underscore the importance of integrating standardized frailty assessments to guide rehabilitation strategies and promote equitable access to multidisciplinary rehabilitation for older adults.

**Supplementary Information:**

The online version contains supplementary material available at 10.1007/s11739-025-04219-4.

## Introduction

The global demographic shift toward population aging has intensified the need for effective strategies to predict and monitor rehabilitation outcomes in older adults. Hospitalized older patients—particularly those admitted to internal medicine wards—often experience a decline in functional autonomy during acute illness, which can prolong length of stay, delay discharge, and increase the risk of institutionalization and readmission [1]. These functional losses, frequently superimposed on frailty and multimorbidity, highlight the importance of identifying early predictors of recovery potential and tailoring rehabilitation interventions accordingly [2, 3].

Standardized clinical scales are widely used in geriatric rehabilitation to evaluate key dimensions of patient function. The Modified Barthel Index measures autonomy in activities of daily living and has shown robust validity, reliability, and interpretability in older populations [4–6]. The Hendrich II Fall Risk Model, validated in acute and subacute care settings, offers a sensitive and specific tool for predicting falls in hospitalized older adults [7, 8]. The Tinetti Scale, also known as performance-oriented mobility assessment, is designed to measure balance and gait function, providing a reliable assessment of mobility [9, 10]. With advancing age, impairments in gait and balance become increasingly prevalent—affecting an estimated 10% of adults aged 60–69 and over 60% of those aged 80 and older [11]. Although these impairments significantly contribute to decreased quality of life, loss of autonomy, and higher fall-related morbidity and mortality [12], evidence indicates that hospital-based geriatric rehabilitation can effectively reduce mortality, the risk of institutionalization, and functional deterioration [13].

Despite the recognized role of chronological age in driving frailty and reduced physiological reserve, its specific influence on recovery trajectories during inpatient rehabilitation remains poorly defined. Emerging evidence suggests that age alone may not be the dominant factor in predicting functional gains, and that baseline status, comorbidities, and cognitive reserve could play more decisive roles [14–16]. Yet, current data are inconsistent, and the extent to which age influences key rehabilitation outcomes across functional domains—such as mobility, autonomy, and fall risk—has not been fully clarified.

This study addresses this knowledge gap by examining the relationship between age and three commonly used geriatric assessment scales—the Modified Barthel Index, Hendrich II Fall Risk Model, and Tinetti Scale—at both admission and discharge in a cohort of hospitalized older adults undergoing rehabilitation. Specifically, we aimed to determine whether age is associated with differences in baseline functional status and in the magnitude of functional recovery.

## Methods

### Patient cohort

This retrospective cohort study included all 159 patients admitted between January 1 and December 31, 2024, to the Cardiorespiratory Rehabilitation Unit of the Pio Albergo Trivulzio Geriatric Hospital in Milan. One patient was excluded because the length of stay was zero, as she was transferred back to the referring hospital on the day of admission due to severe cognitive impairment, which precluded rehabilitation. The final study population therefore consisted of 158 patients. Patients were referred from acute care wards following cardiologic, pneumologic, or neuromotor events, such as heart failure, respiratory failure, pneumonia, or fractures in patients with cardiopulmonary comorbidities. Upon admission, all patients underwent a comprehensive multidimensional geriatric assessment, based on which an individualized rehabilitation program was initiated.

Rehabilitation sessions were conducted daily and included interventions aimed at reducing dyspnea and muscular fatigue, promoting thoracic re-expansion, facilitating bronchial secretion clearance, and reassessing respiratory function through instrumental evaluations. Motor rehabilitation focused on muscle strengthening, balance, and gait recovery. As part of routine clinical practice, the following standardized geriatric assessments were administered at both admission and discharge: the Tinetti Scale, which assesses balance and gait performance [9]; the Hendrich II Fall Risk Model, which identifies fall risk in hospitalized older adults using variables such as confusion, depression, and impaired mobility [7]; and the Modified Barthel Index, which quantifies independence in basic activities of daily living such as feeding, bathing, and ambulation [4].

## Frailty index assessment

Frailty was assessed using a deficit accumulation frailty index (FI), developed according to the methodology proposed by Rockwood and colleagues and operationalized following the framework of Theou et al. [17]. The index was built using 63 variables routinely collected at admission, covering multiple domains relevant to geriatric vulnerability. These included functional abilities (such as continence, transfers, mobility, and personal care), comorbidities involving major organ systems (including cardiovascular, respiratory, renal, hepatic, neurological, musculoskeletal, and endocrine), cognitive function, nutritional status, polypharmacy, and selected laboratory parameters such as hematocrit and serum albumin. Each variable was coded as either “deficit present” or “deficit absent,” and the FI was calculated by dividing the number of present deficits by the total number of considered variables. This yielded a score ranging from 0 (no deficits) to 1 (all deficits present), with higher scores indicating greater frailty. The FI was treated as a continuous variable in the statistical analyses and used as an adjustment covariate to account for biological vulnerability beyond chronological age.

### Ethical approval and informed consent

Ethical approval for this retrospective study (#PAT001) was obtained from the Ethics Committee of the University of Milan on July 8, 2025. Since this was a retrospective study based on routinely collected clinical data, individual informed consent was not required. All data were anonymized at the source, and no information allowing patient identification was collected. The study was conducted in accordance with the ethical principles of the Declaration of Helsinki and all applicable regulatory requirements.

### Statistical analyses

Descriptive statistics are reported as medians with interquartile ranges (IQR) for continuous variables and as frequencies and percentages for categorical variables. Comparisons across age tertiles (T1-age, T2-age, T3-age) were performed using the Kruskal–Wallis test for continuous variables and the Chi-square test for categorical variables. Multivariable linear regression models were built including age, sex, and frailty index as independent variables and Barthel Index, Hendrich II Fall Risk Model, and Tinetti Scale as dependent variables. Additional models tested the role of comorbidity burden (CIRS-IC) and cognitive function (MMSE) as independent variables.

To evaluate age-related differences in functional status and rehabilitation outcomes, patients in the youngest (T1-age) and oldest (T3-age) tertiles were compared. Baseline scores and changes from admission to discharge (delta scores) were analyzed using the nonparametric Mann–Whitney U test. For comparisons at admission, the Modified Barthel and Hendrich II scores were available for 42 patients in the T1-age group and 44 in the T3-age group, while the Tinetti Scale score was available for 30 patients in T1-age and 28 in T3-age. Scores at discharge—and thus the calculation of the delta score—were available for 34 patients in each group for the Modified Barthel score, for 24 patients in T1-age and 31 in T3-age for the Hendrich II score, and for 34 patients in T1-age and 37 in T3-age for the Tinetti Scale. In a secondary analysis, the Tinetti Scale score was further subdivided into its balance and gait components, with 27 patients in T1-age and 23 in T3-age included in each subscale analysis.

We also evaluated whether baseline functional scores predicted post-rehabilitation outcomes using linear regression models with Barthel Index, Hendrich II Fall Risk Model, and Tinetti Scale at admission (T0) as predictors and the corresponding discharge scores (T1) as outcomes. The linearity assumption for continuous predictors was verified through visual inspection of residual plots and comparison of linear and LOESS regression curves. Results are reported as regression coefficients (*β*) with 95% confidence intervals (CI) and *p* values. Statistical significance was denoted as follows: *p < 0.05; **p < 0.01; ***p < 0.001; ****p < 0.0001. All analyses were performed using R statistical software (version 4.4.0).

## Results

### Impact of age on baseline functional status

Among the 158 geriatric patients admitted to the rehabilitation unit, the median age was 84 years (IQR 77–88), with a predominance of female patients (70.8%). The median length of stay was 40 days (IQR 22–67). For comparative analyses, patients were stratified into age tertiles: the youngest group (T1-age) included individuals aged ≤77 years, the intermediate group (T2-age) those aged 78–87 years, and the oldest group (T3-age) those aged ≥88 years. Demographic and clinical characteristics according to age tertiles are presented in Table 1.

**Table 1.** Patient characteristics stratified by age tertiles

Variable	*N*	T1-age	T2-age	T3-age	*p*-value
F/M (%)	158	33/26 (55.9%–44.1%)	39/8 (83.0%–17.0%)	40/12 (76.9%–23.1%)	0.0049
Age in years, median [IQR]	158	76.0[71.0–78.0]	84.0[82.0–85.0]	90.0[88.0–92.0]	<0.0001
Length of stay, median [IQR]	158	35.0[20.5–58.5]	42.0[31.0–69.0]	43.0[31.0–76.5]	0.0536
MMSE score, median [IQR]	120	26.0[23.0–29.0]	24.1[21.0–27.0]	23.4[19.6–25.0]	0.0095
BRASS, median [IQR]	156	19.0[15.0–21.0]	22.0[20.0–24.0]	23.0[21.0–25.0]	<0.0001
CIRS IC, median [IQR]	152	2.1[1.9–2.3]	2.1[1.9–2.2]	2.0[1.9–2.3]	0.7263
CIRS IS, median [IQR]	152	5.0[4.0–7.0]	6.0[5.0–7.0]	5.0[3.5–6.0]	0.3500
Hendrich II score, median [IQR]	158	4.0[2.0–6.0]	5.0[4.0–7.0]	5.0[4.0–7.0]	0.0360
Barthel Index, median [IQR]	158	32.0[15.5.–64.5]	19.0[13.5–25.5]	16.0[7.5–30.0]	0.0002
Tinetti scale, median [IQR]	152	11.0[2.0–17.5]	8.0[0.2–12.8]	5.0[0.0–10.5]	0.0544
Polypharmacy (>5 drugs, %)	97	30 (96.8%)	32 (97.0%)	32 (97.0%)	1.0000
Frailty Index	158	0.6[0.4–0.7]	0.7[0.6–0.7]	0.7[0.6–0.8]	0.1007

*MMSE* Mini-Mental State Examination, *BRASS* Blaylock Risk Assessment Screening Score, *CIRS-IC* Cumulative Illness Rating Scale—Index of Comorbidity, *CIRS-IS* Cumulative Illness Rating Scale—Index of Severity

Female sex was more prevalent in older age groups. Cognitive function, assessed with the MMSE, progressively declined with age (median 26.0 in T1 vs. 23.4 in T3; *p* = 0.0095), and BRASS scores increased, indicating greater discharge complexity (median 19.0 in T1 vs. 23.0 in T3; *p* < 0.0001). Comorbidity burden (CIRS-IC and CIRS-IS) and frailty index did not significantly differ across age groups, as well as the frailty index (median 0.6 in T1 vs. 0.7 in T3; *p* = 0.1007). Functional status at admission worsened with increasing age, with higher Hendrich II scores (median 4.0 in T1 vs. 5.0 in T3; *p* = 0.0360), lower Barthel Index scores (median 32.0 in T1 vs. 16.0 in T3; *p* = 0.0002), and lower Tinetti Scale scores (median 11.0 in T1 vs. 5.0 in T3; *p* = 0.0544). Polypharmacy (defined as the use of >5 drugs) was highly prevalent and comparable across age groups.

Sex stratification of the patient population showed that females were significantly older (median 84 vs 79 years, *p* = 0.0045) and exhibited higher frailty index scores (0.7 vs 0.6, *p* = 0.0165). Women also showed lower Barthel Index and Tinetti Scale scores, indicating reduced functional autonomy and mobility (*p* = 0.0052 and *p* = 0.0035, respectively). No sex differences were observed in MMSE, CIRS-IC, CIRS-IS, or Hendrich II scores (Supplementary Table 1).

### Association of age with baseline functional parameters

To better understand the impact of chronological age on functional status at admission, we examined its independent association with performance scores after adjusting for sex and frailty index. In multivariable linear regression models adjusted for sex and frailty index, age was independently associated with Barthel scores (*β* =  −0.65 per year; 95% CI −0.93 to −0.36; *p* < 0.001), but not with Tinetti or Hendrich scores. Frailty index showed a strong and consistent association with all functional outcomes: Tinetti (*β* =  −39.9, *p* < 0.001), Barthel (*β* =  −89.9, *p* < 0.001), and Hendrich (*β* = 6.8, *p* < 0.001). Sex was not significantly associated with any functional score (Table 2).

**Table 2.** Multivariable linear regression analysis evaluating the association between age, sex, and frailty index and functional outcomes (Tinetti scale, Barthel Index, and Hendrich II score) at hospital admission

Outcome	Term	Estimate (*β*)	95% CI	*p*-value
Tinetti scale	Age	−0.023	[−0.114, 0.068]	0.616
	Sex	0.342	[−1.244, 1.927]	0.671
	Frailty index	−39.876	[−44.194, −35.557]	1.04e–39
Barthel Index	Age	−0.647	[−0.933, 0.360]	1.60e–05
	Sex	2.17	[−2.827, 7.167]	0.392
	Frailty index	−89.901	[−103.515, −76.288]	2.18e–26
Hendrich II score	Age	0.017	[−0.035, 0.068]	0.526
	Sex	0.337	[−0.560, 1.234]	0.459
	Frailty index	6.827	[4.383, 9.271]	1.48e–07

When comorbidity burden (CIRS-IC) was included as an independent variable in the model, with frailty index and sex as covariates, it was significantly associated only with Tinetti scores (*β* = 8.51, 95% CI 5.73–11.30, *p* = 1.9 × 10⁻⁸), indicating that higher comorbidity burden predicted poorer balance and gait performance (Supplementary Table 2). Cognitive function (MMSE), entered as an independent variable with the same covariates, was not significantly associated with any of the functional outcomes (Supplementary Table 3).

### Impact of age on rehabilitation outcomes

**Fig. 1:**
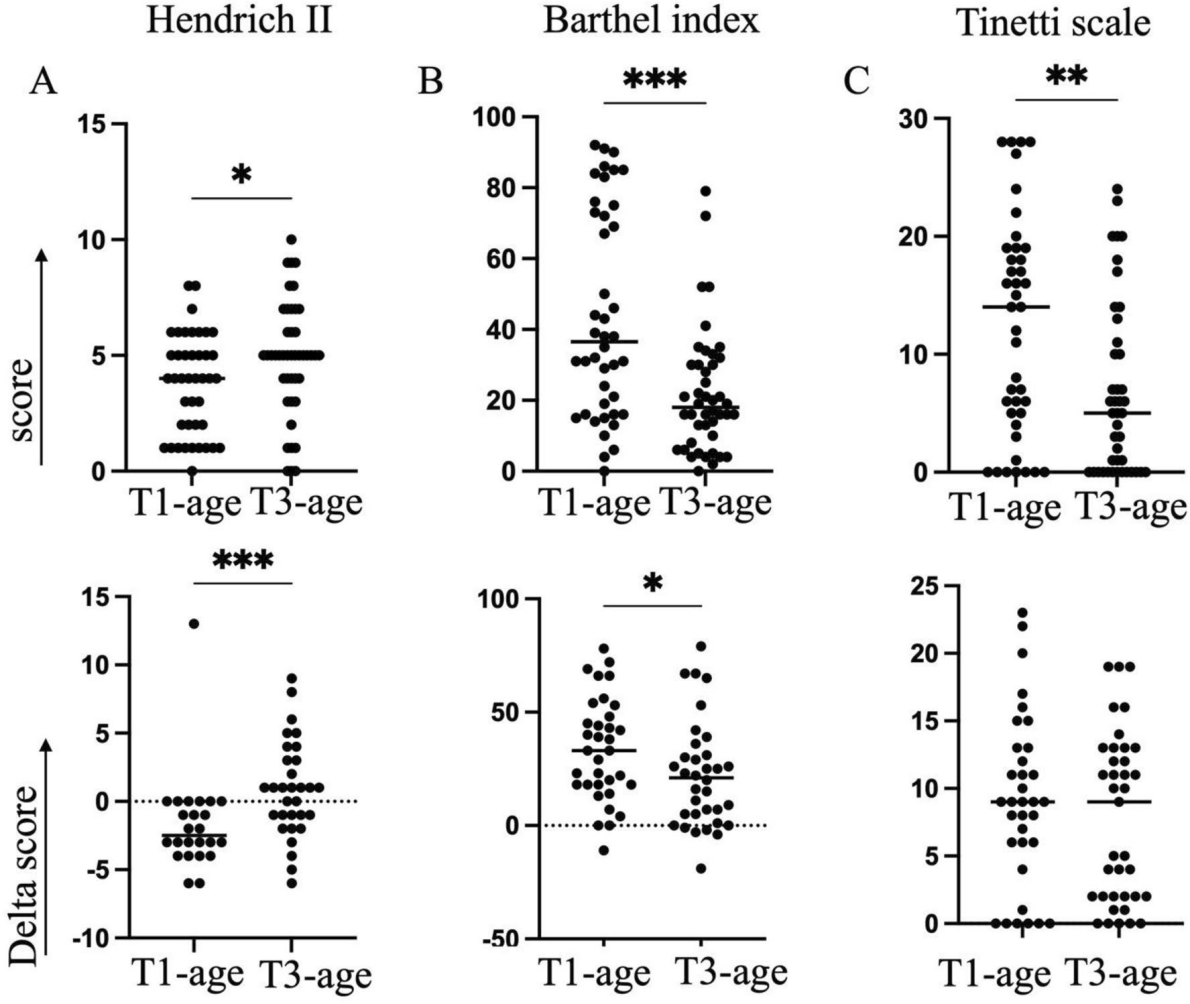
Geriatric assessment scores at hospital admission and changes during rehabilitation by age tertiles. Panels A–C (upper): Baseline scores at admission for the Hendrich Fall Risk Model (A), Barthel Index (B), and Tinetti Scale, sum of balance and gait, (C) in the youngest (T1-age) and oldest (T3-age) tertiles. Panels A–C (lower): changes in scores from admission to discharge (Delta score = discharge—admission) for each assessment tool in the T1-age and T3-age groups. Each dot represents an individual patient. Horizontal dashed lines indicate no change following rehabilitation. *p* values were calculated using the Mann–Whitney U test

To evaluate whether age influenced rehabilitation trajectories, we compared functional outcomes between the youngest (T1-age) and oldest (T3-age) tertiles. As expected, the T3-age group showed significantly worse baseline scores across all domains (Fig. 1A–C, upper panels), confirming that advanced age was associated with higher functional impairment, increased fall risk, and reduced autonomy and mobility at admission.

Analysis of score changes from admission to discharge revealed age-dependent responses to rehabilitation (Fig. 1A–C, lower panels). At discharge, Hendrich II scores remained elevated in the T3-age group compared to T1-age patients, indicating a persistently high fall risk despite the completion of rehabilitation (Fig. 1A, lower panel). Although Modified Barthel Index scores improved in both groups, the magnitude of improvement was significantly smaller in the T3-age group, suggesting more limited recovery of functional autonomy (Fig. 1B, lower panel). In contrast, Tinetti Scale scores improved substantially and to a similar extent in both age groups, indicating that mobility is responsive to rehabilitation regardless of age (Fig. 1C, lower panel). To further assess the impact of age on motor recovery, we analyzed the two subcomponents of the Tinetti Scale: balance and gait. At admission, patients in the T3-age group exhibited significantly lower scores in both domains compared to those in the T1-age group, with a median balance score of 5 versus 8.5 and a median gait score of 2 versus 7.5, respectively, indicating marked impairment, particularly in gait function (Supplementary Fig.  1 A, B). By discharge, both balance and gait scores improved significantly in both groups. Notably, the improvement in gait performance was particularly pronounced, with a median delta of 3.8 in T1-age and 4.5 in T3-age, suggesting that age did not limit the capacity for gait recovery.

### Baseline functional scores predict post-rehabilitation outcomes

Baseline functional scores (at admission) were tested as predictors of post-rehabilitation outcomes (Supplementary Table 4). Higher Tinetti scores at admission were significantly associated with higher scores at discharge (*β* = 0.58, 95% CI 0.45–0.71, *p* = 5.6 × 10⁻^15^). Similarly, higher Barthel Index scores at baseline strongly predicted better functional recovery (*β* = 0.87, 95% CI 0.68–1.06, *p* = 5.3 × 10⁻^15^). Baseline Hendrich II scores were also significantly associated with post-rehabilitation scores, although with a weaker effect size (*β* = 0.31, 95% CI 0.08–0.54, *p* = 0.0095). These findings highlight the central role of baseline functional status in predicting rehabilitation outcomes.

### Assessing malnutrition as a determinant of fall risk

By the end of the rehabilitation program, the risk of falls—as measured by the Hendrich II score—remained significantly higher in the older cohort. To investigate potential contributors to this persistent vulnerability, we examined whether malnutrition could explain the incomplete recovery of fall risk. Nutritional status at admission was assessed using the Malnutrition Universal Screening Tool (MUST) [18]. Results were comparable between age groups: only three patients in each group had a MUST score ≥2, indicating high risk of malnutrition (Supplementary Fig. 2). These findings suggest that malnutrition alone is unlikely to explain the sustained fall risk observed in the oldest patients.

## Discussion

This study demonstrates that while older age is associated with poorer baseline functional status, it does not significantly limit motor recovery following hospital rehabilitation. In contrast, frailty emerged as the strongest and most consistent determinant of functional outcomes. Specifically, the frailty index showed robust associations with all functional scores, whereas chronological age was independently associated only with the Barthel Index and had no significant effect on Tinetti or Hendrich II scores. These findings indicate that biological vulnerability, captured by frailty, rather than chronological age per se, better explains the variability in rehabilitation trajectories among older adults. Notably, patients in the oldest age tertile achieved similar gains in Tinetti scores compared to younger individuals, highlighting a preserved capacity for motor recovery with advancing age. This challenges age-based assumptions and supports an inclusive approach to rehabilitation that prioritizes individualized assessment over age as a criterion for access to care. The World Health Organization has emphasized the need to combat ageism to ensure equitable access to care and improve the health of older populations [19, 20]. The concept of frailty provides a more accurate framework to stratify patients and predict rehabilitation potential. Frailty reflects reduced physiological reserve and increased vulnerability to stressors, and has been consistently shown to be a stronger predictor of adverse outcomes than chronological age [21], [22, 23]. Tools such as the Fried phenotype [24] or Rockwood’s frailty index [25] provide validated approaches for frailty assessment and may be particularly useful in rehabilitation settings to guide treatment intensity, resource allocation, and discharge planning.

Despite substantial improvements in mobility across all age groups, recovery of functional autonomy and fall risk was more limited in older patients. The Barthel Index and Hendrich II score improved less than the Tinetti Scale, particularly in the oldest age group. This likely reflects the multidimensional nature of these outcomes. The Barthel Index includes domains such as feeding, hygiene, and sphincter control that are influenced by cognitive, emotional, and social factors—not only motor function. Similarly, the Hendrich II score incorporates age-related factors such as confusion, incontinence, sedative use, and impaired mobility [26–29], which are less responsive to physical rehabilitation alone. Moreover, the Hendrich II score does not account for extrinsic fall risk factors—such as environmental hazards, lighting, or footwear—which may also contribute to persistent vulnerability.

We also explored the potential contribution of malnutrition, a key driver of frailty and functional decline. Nutritional status at admission, assessed using the MUST tool, was similar across age groups, suggesting that malnutrition alone does not explain the reduced improvement in fall risk among older patients. Malnutrition is a clinical marker often associated with sarcopenia [30]. However, specific assessments of sarcopenia (e.g., muscle mass or strength) were not available in this cohort. Sarcopenia, defined as the loss of muscle mass and function, is a major determinant of disability in older adults and should be routinely assessed alongside nutritional status for a more comprehensive evaluation [30].

The strong predictive value of baseline functional scores for post-rehabilitation outcomes underscores the importance of early assessment upon admission. Patients with better functional status at baseline achieved greater improvements at discharge, emphasizing the role of early mobilization and prevention of pre-hospital functional decline to optimize rehabilitation outcomes.

This study has several limitations. First, its retrospective and single-center design may limit the generalizability of findings. Second, adherence to the rehabilitation program was not systematically recorded, preventing evaluation of its impact on outcomes. Third, the assessment tools used, although standardized and widely validated, may not fully capture the complexity of functional trajectories in older adults. For example, the Hendrich II Fall Risk Model may underestimate the contribution of extrinsic factors, while the Barthel Index may not capture improvements in non-motor domains. Finally, the lack of sarcopenia measures is another limitation.

Taken together, these findings highlight the importance of a multidimensional and individualized approach to geriatric rehabilitation. Frailty—not chronological age—should guide clinical decision-making, resource allocation, and rehabilitation goals. Interventions should address not only mobility but also cognitive, nutritional, pharmacological, and environmental factors influencing functional decline and fall risk [31]. In this context, the Tinetti Scale emerged as the most sensitive indicator of motor improvement, while the Barthel and Hendrich scales captured broader and more complex domains of autonomy and vulnerability. These results align with the WHO ICOPE framework, which emphasizes maintaining intrinsic capacity and addressing frailty to promote healthy aging and functional independence [31].

## Conclusions

This study demonstrates that chronological age alone is not a limiting factor for rehabilitation outcomes in older adults. Although older patients present with greater functional impairment at admission, their potential for motor recovery remains substantial, particularly with respect to mobility. In contrast, frailty emerged as a stronger determinant of functional outcomes than age per se, underscoring the need to move beyond age-based decision-making.

These findings highlight the importance of integrating standardized frailty assessments into clinical practice to better identify patients at risk of poorer outcomes and to tailor rehabilitation strategies accordingly. Future prospective, multicenter studies with comprehensive adjustment for clinical covariates are needed to validate and extend these observations.

## Supplementary Information

Below is the link to the electronic supplementary material.

Supplementary file1 (DOCX 413 KB)

## Data Availability

The datasets analyzed during the current study are not publicly available due to ethical and privacy restrictions, but are available from the corresponding author on reasonable request.

## References

[1] Ceriani E et al (2025) COmplexity of CARE and discharge barriers: the ‘modern internal medicine patient’. Results from the CO-CARED study. Intern Emerg Med 20(2):471–479 39656348 10.1007/s11739-024-03823-0

[2] Bunn JG et al (2025) Approaches to characterising multimorbidity in older people accessing hospital care: a scoping review. Eur Geriatr Med. 10.1007/s41999-025-01166-3 40025289 10.1007/s41999-025-01166-3 PMC12378491

[3] Pilotto A et al (2025) A digital-health program based on comprehensive geriatric assessment for the management of older people at their home: final recommendations from the MULTIPLAT_AGE network project. Healthcare. 10.3390/healthcare13101105 40427942 10.3390/healthcare13101105 PMC12111062

[4] Hsieh YW et al (2007) Establishing the minimal clinically important difference of the Barthel Index in stroke patients. Neurorehabil Neural Repair 21(3):233–238 17351082 10.1177/1545968306294729

[5] Granger CV, Albrecht GL, Hamilton BB (1979) Outcome of comprehensive medical rehabilitation: measurement by PULSES profile and the Barthel Index. Arch Phys Med Rehabil 60(4):145–154 157729

[6] Castiglia SF et al (2017) The culturally adapted Italian version of the Barthel Index (IcaBI): assessment of structural validity, inter-rater reliability and responsiveness to clinically relevant improvements in patients admitted to inpatient rehabilitation centers. Funct Neurol 22(4):221–228 29306359 10.11138/FNeur/2017.32.4.221 PMC5762108

[7] Hendrich AL, Bender PS, Nyhuis A (2003) Validation of the Hendrich II fall risk model: a large concurrent case/control study of hospitalized patients. Appl Nurs Res 16(1):9–21 12624858 10.1053/apnr.2003.YAPNR2

[8] Loenneke JP et al (2017) Time-course of muscle growth, and its relationship with muscle strength in both young and older women. Geriatr Gerontol Int 17(11):2000–2007 28276188 10.1111/ggi.13010

[9] Tinetti ME (1986) Performance-oriented assessment of mobility problems in elderly patients. J Am Geriatr Soc 34(2):119–126 3944402 10.1111/j.1532-5415.1986.tb05480.x

[10] Faber MJ, Bosscher RJ, van Wieringen PC (2006) Clinimetric properties of the performance-oriented mobility assessment. Phys Ther 86(7):944–954 16813475

[11] Salzman B (2010) Gait and balance disorders in older adults. Am Fam Physician 82(1):61–68 20590073

[12] Ambrose AF, Paul G, Hausdorff JM (2013) Risk factors for falls among older adults: a review of the literature. Maturitas 75(1):51–61 23523272 10.1016/j.maturitas.2013.02.009

[13] Wong EKC et al (2024) Effectiveness of geriatric rehabilitation in inpatient and day hospital settings: a systematic review and meta-analysis. BMC Med 22(1):551 39578865 10.1186/s12916-024-03764-7 PMC11583748

[14] Bagg S, Pombo AP, Hopman W (2002) Effect of age on functional outcomes after stroke rehabilitation. Stroke 33(1):179–185 11779908 10.1161/hs0102.101224

[15] Shi S et al (2022) Analysis of functional recovery in older adults discharged to skilled nursing facilities and then home. JAMA Netw Open 5(8):e2225452 36006647 10.1001/jamanetworkopen.2022.25452 PMC9412223

[16] McCusker J, Kakuma R, Abrahamowicz M (2002) Predictors of functional decline in hospitalized elderly patients: a systematic review. J Gerontol A Biol Sci Med Sci 57(9):M569–M577 12196493 10.1093/gerona/57.9.m569

[17] Theou O et al (2023) How to construct a frailty index from an existing dataset in 10 steps. Age Ageing. 10.1093/ageing/afad221 38124255 10.1093/ageing/afad221 PMC10733590

[18] Stratton RJ et al (2004) Malnutrition in hospital outpatients and inpatients: prevalence, concurrent validity and ease of use of the ‘malnutrition universal screening tool’ (‘MUST’) for adults. Br J Nutr 92(5):799–808 15533269 10.1079/bjn20041258

[19] https://www.who.int/teams/social-determinants-of-health/demographic-change-and-healthy-ageing/combatting-ageism/global-report-on-ageism.

[20] Burnes D et al (2019) Interventions to reduce ageism against older adults: a systematic review and meta-analysis. Am J Public Health 109(8):e1–e9 31219720 10.2105/AJPH.2019.305123 PMC6611108

[21] Hoenig H, Nusbaum N, Brummel-Smith K (1997) Geriatric rehabilitation: state of the art. J Am Geriatr Soc 45(11):1371–1381 9361665 10.1111/j.1532-5415.1997.tb02939.x

[22] Proietti M, Cesari M (2020) Frailty: what is it? Adv Exp Med Biol 1216:1–7 31894541 10.1007/978-3-030-33330-0_1

[23] Mitnitski AB, Mogilner AJ, Rockwood K (2001) Accumulation of deficits as a proxy measure of aging. Scientific World J 1:323–336 10.1100/tsw.2001.58 PMC6084020 12806071

[24] Pritchard JM et al (2017) Measuring frailty in clinical practice: a comparison of physical frailty assessment methods in a geriatric out-patient clinic. BMC Geriatr 17(1):264 29132301 10.1186/s12877-017-0623-0 PMC5683585

[25] Rockwood K, Hogan DB, MacKnight C (2000) Conceptualisation and measurement of frailty in elderly people. Drugs Aging 17(4):295–302 11087007 10.2165/00002512-200017040-00005

[26] Hofer-Duckelmann C (2012) Gender and polypharmacotherapy in the elderly: a clinical challenge. Handb Exp Pharmacol 214:169–182 10.1007/978-3-642-30726-3_9 23027451

[27] Jaqua EE, Nguyen VTN, Chin E (2023) Delirium in older persons: prevention, evaluation, and management. Am Fam Physician 108(3):278–287 37725462

[28] Enck P et al (1989) Age and sex and anorectal manometry in incontinence. Dis Colon Rectum 32(12):1026–1030 2591277 10.1007/BF02553874

[29] Milsom I, Gyhagen M (2019) The prevalence of urinary incontinence. Climacteric 22(3):217–222 30572737 10.1080/13697137.2018.1543263

[30] Cruz-Jentoft AJ et al (2019) Sarcopenia: revised European consensus on definition and diagnosis. Age Ageing 48(4):601 31081853 10.1093/ageing/afz046 PMC6593317

[31] Cesari M et al (2022) Implementing care for healthy ageing. BMJ Glob Health. 10.1136/bmjgh-2021-007778 35185014 10.1136/bmjgh-2021-007778 PMC8860009

