# Supplementary material for: Impact of age on functional recovery following hospital-based rehabilitation in older adults
Source: Intern Emerg Med. 2025 Dec 14;21(2):569–76. doi: 10.1007/s11739-025-04219-4 (PMC13061775; doi:10.1007/s11739-025-04219-4)
Supplement: Supplementary file 1 — Supplementary file1 (DOCX 413 KB) [file 11739_2025_4219_MOESM1_ESM.docx]

**Supplementary material**

**Supplementary Tables**

|  |
| --- |
| Supplementary Table 1. Comparison of functional outcomes at admission between male and female patients. |

|  |
| --- |
| Supplementary Table 2. Multivariable linear regression analysis assessing the association between **CIRS-IC (comorbidity burden)**, sex, and frailty index with functional outcomes (**Tinetti scale**, **Barthel Index**, and **Hendrich II score)** at admission. |

|  |
| --- |
| Supplementary Table 3. Multivariable linear regression analysis assessing the association between **MMSE (cognitive function)**, sex, and frailty index with functional outcomes (**Tinetti scale**, **Barthel Index**, and **Hendrich II score)** at admission. |

|  |
| --- |
| Supplementary Table 4. Linear regression analysis evaluating the association between baseline **Tinetti scale**, **Barthel Index**, and **Hendrich II score** at admission (**T0**) and functional outcomes at discharge (**T1**). |

**Supplementary Figures**


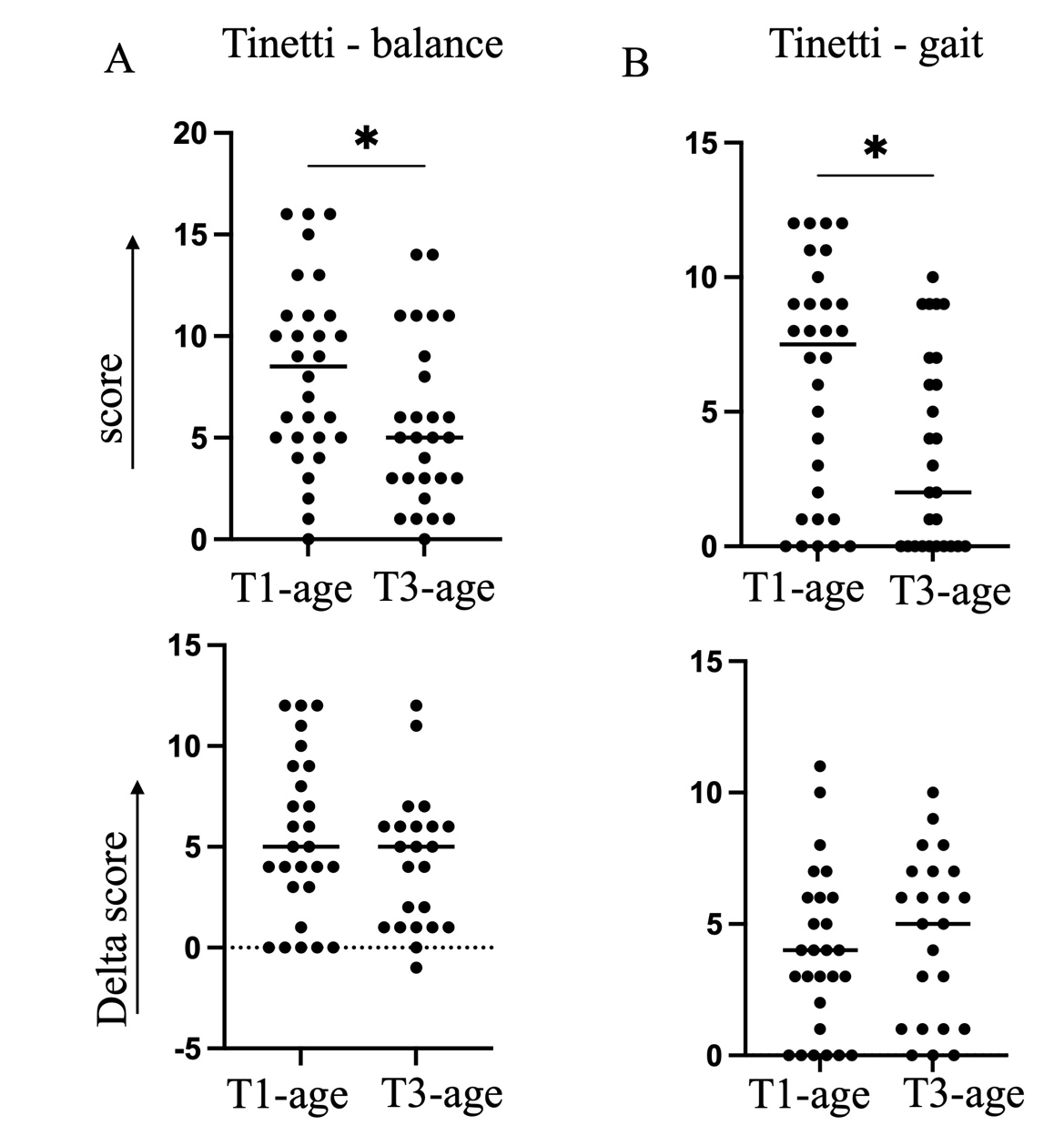


**Supplementary Figure 1. Tinetti balance and gait scores at hospital admission and changes during rehabilitation by age tertiles.** Panels A–B (upper): Baseline scores at admission for the Tinetti Scale - Balance (A), Tinetti Scale - Gait (B) in the youngest (T1-age) and oldest (T3-age) tertiles. Panels A–B (lower): Changes in scores from admission to discharge (Delta score = discharge – admission) in the T1-age and T3-age groups. Each dot represents an individual patient. Horizontal dashed lines indicate no change following rehabilitation. p values were calculated using the Mann–Whitney U test.


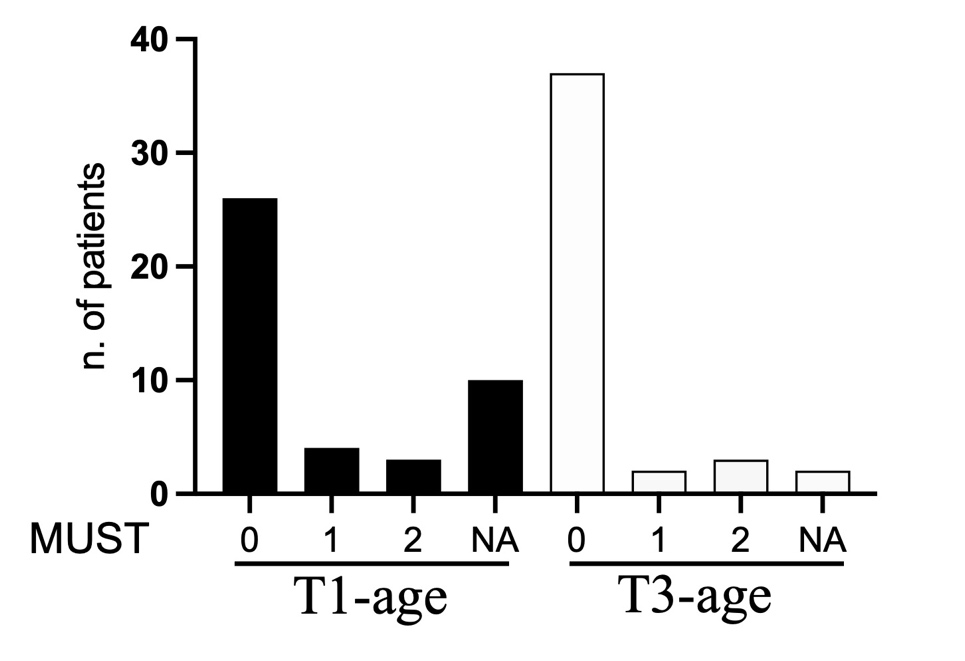


**Supplementary Figure 2.** Distribution of the MUST score in the patient population based on age tertiles (youngest, T1-age and oldest, T3-age tertiles).
